# Role of NMDA receptor subtypes in different forms of NMDA-dependent synaptic plasticity

**DOI:** 10.1186/1471-2202-8-55

**Published:** 2007-07-26

**Authors:** Rui Li, Fen-Sheng Huang, Abdul-Karim Abbas, Holger Wigström

**Affiliations:** 1 Department of Medical Biophysics, Institute of Neuroscience and Physiology, Göteborg University, Box 433, 405 30 Göteborg, Sweden

## Abstract

**Background:**

The involvement of different NMDA receptor (NMDAR) subunits has been implicated in several forms of synaptic plasticity. However, it is still controversial to what extent the involvement is specific, and little is known about the role of NMDAR subunits in certain "non-conventional" forms of plasticity. In this study we used subunit-specific blockers to test the roles of NR2A- and NR2B-containing NMDARs in a type of chemical long-term depression (LTD) induced by brief bath application of the NMDAR agonist NMDA to hippocampal slices from 12–18 days old rats. For comparison, we also examined other forms of plasticity, including a "slow LTD" induced by 0.1 Hz stimulation under low Mg^2+ ^conditions as well as long-term potentiation (LTP).

**Results:**

A blocker of NR2A-containing NMDARs, NVP-AAM077 (NVP), substantially reduced the two forms of studied depression whereas blockers of NR2B-containing NMDARs, Ro25-6981 (Ro) or Ifenprodil (Ife), had no significant effect on them. LTP appeared to be more sensitive as it was fully blocked by NVP and partially blocked by Ro or Ife. However, the blocking effects of NVP could be counteracted by general amplification of NMDA responses by lowering Mg^2+ ^concentration in the perfusion solution. Applying NVP or Ro/Ife on isolated NMDA-EPSPs recorded in low Mg^2+ ^solution reduced responses to about 70% and 20% of initial size, respectively, whereas coapplication of both blockers almost completely abolished the responses. Additionally, NMDA application caused depotentiation of a pathway with prior tetanus-induced LTP, and NVP but not Ro/Ife substantially prevented that depotentiation as well as the chemical LTD of the control pathway. A second tetanus on the LTP pathway induced repotentiation which was fully blocked by NVP but partially blocked by Ro/Ife.

**Conclusion:**

All of these results on hippocampal slices from young rats can be explained by a simple model, in which NR2A subunits dominate over NR2B subunits with respect to both plasticity and NMDAR-mediated responses. The model suggests that Ca^2+ ^influx into the postsynaptic spine via different subtypes of NMDARs makes up a "final common pathway", controlling synaptic plasticity by its magnitude and temporal pattern regardless of the source.

## Background

Long-term potentiation (LTP) and long-term depression (LTD) are forms of activity-dependent synaptic plasticity believed to play important roles in learning and memory processes. Experimentally, LTP is induced by brief high frequency activation of presynaptic axons (e.g. 100 Hz, 1 s) or by pairing presynaptic activation with postsynaptic depolarization, resulting in a long-lasting increase of the synaptic efficacy [1-4]. LTD, on the other hand requires activation at a moderate frequency (e.g. 1 Hz, 15 min) or paring presynaptic activation with lower levels of depolarization, leading to a long-lasting decrease in synaptic efficacy [5,6]. Both types of plasticity depend on NMDA receptor (NMDAR) activation as a first step, evidenced by the fact that they are fully blocked when NMDAR antagonists, such as AP5, are present during the induction. Additionally, in many brain regions synaptic potentiation as well as depression can be induced by direct application of NMDA, the specific agonist of NMDAR. While fast application of NMDA, e.g. by pressure injection or iontophoresis, may lead to LTP-like phenomena [7,8], bath application of NMDA for several minutes has been shown to induce a persistent depression, believed to be equivalent to stimulus-induced LTD [9]. Our previous work on NMDA-induced plasticity has demonstrated multiple changes after NMDA application, some of which may be related to LTP/LTD while others may represent different forms of synaptic plasticity [10].

NMDAR are tetrameric, or possibly pentameric, complexes containing at least one NR1 receptor subunit and two or more NR2 units [11-13]. NR1 subunits distribute ubiquitously in the mammalian central nervous system and are required for receptor function [14]. NR2 subunits exist in different isoforms NR2A, NR2B, NR2C and NR2D. The composition of the receptor in terms of these units determines some important properties of the receptor, including kinetics, open channel conductance and voltage dependence [14]. The balance between 2A and 2B changes during development, with NR2B-type NMDARs being prominent in young animals while NR2A-type NMDARs become more important with increasing age [15,16]. A critical issue is to what extent the different NMDAR subunits are involved in different forms of NMDA-dependent synaptic plasticity such as LTP and LTD. Whereas an early study suggested a difference between NR2A-B on one hand and NR2C-D on the other hand [17], later work has emphasized the distinction between NR2A and NR2B. Thus, it has been shown that LTP in the hippocampus is specifically related to NR2A-containing NMDARs [18]. On the other hand, LTD in both hippocampus and perirhinal cortex was shown to specifically rely on NR2B-containing NMDARs [18,19]. Moreover, it was reported that the erasure of LTP, referred to as "depotentiation", was due to the involvement of NR2A subunits [19]. Even so, the idea of subtype specificity has also been questioned, suggesting that the important thing for LTP is the degree and temporal character of the Ca^2+ ^influx, not the actual subtype of receptor involved [20]. Studies in some other brain regions than hippocampus, such as anterior cingulate cortex (ACC), also showed that both NR2A- and NR2B-type NMDARs contributed to LTP [21].

As shown above, data on subunit specificity exist for processes such as LTP and LTD as well as depotentiation, the "erasure counterpart" of LTP. However, little is known about the role of NMDAR subtypes in synaptic plasticity induced by direct application of NMDA. Some previous work studied the immediate effects of NMDA in terms of depolarization or the associated depression of EPSPs in connection with subunit-specific blockers, but the concentration of NMDA in those cases was usually much smaller than needed to get persistent depression of EPSPs [22,23]. We were therefore eager to study the issue of NMDAR subunit specificity in connection with the above-mentioned LTD model, using NMDA application instead of stimulation as the induction event. The results suggested an essential role for NR2A- rather than NR2B-type NMDARs and were further complemented by measurements on isolated NMDA-EPSPs. This prompted us to investigate other plasticities such as LTP, depotentiation and a form of stimulus-induced, slowly developing LTD. Taken together, our results imply a predominant NR2A-dependency in all of the cases. However, it appeared that NR2B subunits also contributed under certain conditions.

## Results

In the first set of experiments, we examined the effects of selective antagonists of NR2A and NR2B subunits on NMDA-induced plasticity. While a previous study in this lab has revealed multiple changes induced by NMDA application, some of which are transient, here we were mainly interested in the effects on the sustained depression obtained about one hour after NMDA treatment. This plasticity will be referred to as NMDA-induced LTD in the following, as it is believed to be equivalent to stimulus-induced LTD. As blockers we used NVP-AAM077 (NVP) for NR2A-containing NMDARs and either Ro25-6981 (Ro) or Ifenprodil (Ife) for NR2B-containing NMDARs (see Methods).

### NMDA-induced LTD

**Figure 1:**
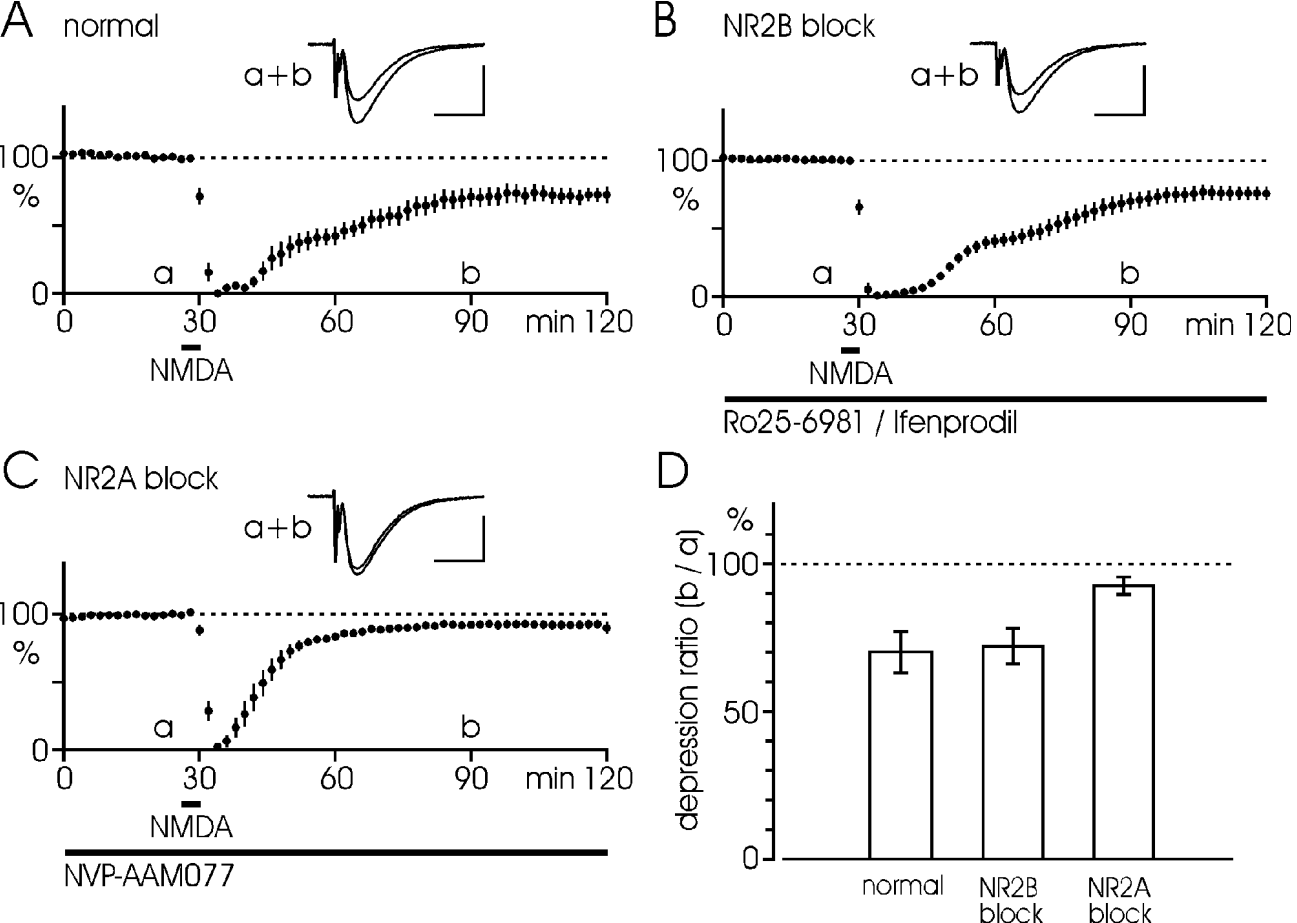
**Roles of NR2B and NR2A subunits in NMDA-induced LTD**. (A) NMDA application (30 μM, 4 min) under normal conditions causes initial extinction of EPSPs, followed by recovery and stabilization (n = 15). (B) NR2B inhibitors, Ro25-6981 (0.5 μM) or Ifenprodil (3 μM), do not affect NMDA-induced plasticity (n = 8+4 = 12). (C) In presence of NR2A inhibitor, NVP-AAM077 (0.4 μM), NMDA effects are different compared with control situation, with a shorter recovery phase and much less depression (n = 10). (D) Bar diagram reveals LTD at 60 min after NMDA application as percentage of the initial baseline under the different experimental conditions in A-C. Data are given as mean ± S.E.M. Black bars in A-C indicate the duration of drug treatment. Values are shown averaged for 2 min periods. Inserts illustrate EPSP-traces taken at the indicated time points (a, b). Calibrations: 0.5 mV, 10 ms.

NVP (0.4 μM) or Ro (0.5 μM)/Ife (3 μM) was applied at least 1 h before NMDA application and no influence on baseline level was observed. Fig. 1 illustrates NMDA-induced LTD under normal conditions compared to similar experiments treated with NVP or Ro/Ife. The control experiment in Fig. 1A shows that brief (4 min) application of NMDA (30 μM) resulted in a complete extinction of EPSPs, followed by a recovery and a stable, depressed level about 1 h later, i.e. NMDA-induced LTD. On average, EPSPs were depressed to 70 ± 7%, n = 15, of the initial baseline level. As seen in Fig. 1B, blockade of NR2B receptors by Ro/Ife did not cause any detectable change in NMDA-induced plasticity as revealed by a nearly identical time course of the depression. In this case, EPSPs were depressed to 72 ± 6% (n = 12) of baseline, which is almost the same as in the control situation (p > 0.1, see also bar diagram in Fig. 1D). Fig. 1C shows the situation in the presence of NVP, yielding a different result. It can be seen that the extinction phase was shorter with about 10 min to half recovery compared to about 30 min in the normal case. Moreover, the LTD was nearly blocked by this NR2A inhibitor, amounting to 92 ± 3% (n = 10, p < 0.05) of baseline. Bar diagram in Fig. 1D summarizes the quantitative data.

### Isolated NMDA-EPSPs

**Figure 2:**
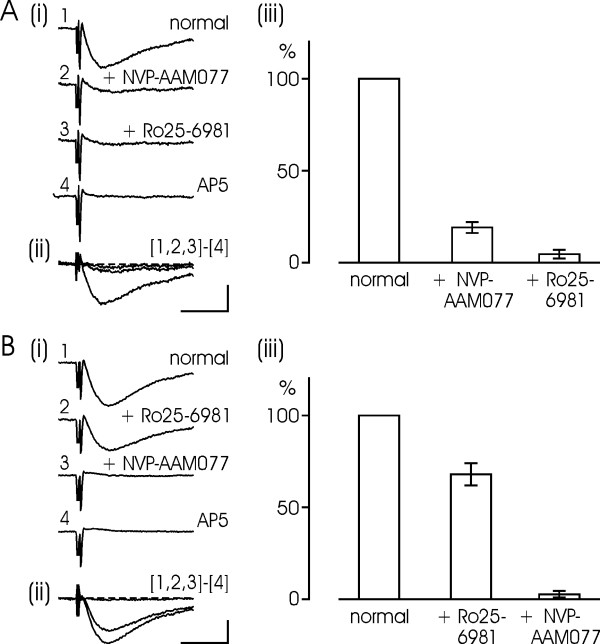
**Relative contributions of NR2B and NR2A subunits to isolated NMDA-EPSPs**. (A) Isolated NMDA-EPSPs obtained in a low Mg^2+ ^solution (0.1 mM; n = 5). (i) After recording baseline responses, defining the 100% level (1), applying NR2A inhibitor NVP-AAM077 (0.4 μM) leads to a substantial reduction of the isolated NMDA response (2). Adding NR2B inhibitor Ro25-6981 (0.5 μM) further depresses the responses down to near zero (3). Subsequent perfusion with AP5 (50 μM) fully blocks the synaptic responses and values obtained in this solution are taken as zero level (4). (ii) Traces 1–3 plotted together after subtraction of the zero level. (iii) Bar diagram quantifying the reductions of NMDA-EPSPs after sequentially adding the two subunit-specific blockers, NVP-AAM077 and Ro25-6981. (B) Similar plots for another set of experiments where the two blockers were applied in a different order (n = 6). The bar diagrams show data as mean ± S.E.M. Calibrations: 0.1 mV, 20 ms.

Isolated NMDAR-mediated EPSPs were recorded with the help of low Mg^2+ ^solution. Since frequent activation of NMDARs can cause a successive decrease of the response amplitude, the stimulus frequency was kept low, usually at one per minute. After recording such NMDA-EPSPs for 30–60 min, the NR2A antagonist NVP (0.4 μM) was applied leading to a substantial reduction of responses, on the average down to 19 ± 3% of baseline (n = 5). Subsequent application of Ro (0.5 μM) depressed the responses further down to 4.6 ± 2.4% of the original baseline. Finally, AP5 (50 μM) solution, assumed to block all types of NMDARs, was applied. The response in AP5 was used to define the zero response (see Methods for the concept of the "nonsynaptic potential"). It appears that NR2A- and NR2B-type NMDARs mediate most of the NMDA-EPSP, with the dominance of NR2A-containing receptors (see Fig. 2A). Our results were corroborated by alternative experiments in which the same concentrations of drugs were applied but in a different order. Thus we first applied Ro, then NVP and finally switch the perfusion to AP5 solution (Fig. 2B). It was found that Ro caused a partial reduction of the NMDA response, down to 68 ± 6% of baseline (n = 6), whereas subsequent application of NVP blocked virtually all of the response, down to 2.7 ± 1.8% of baseline. Similar results were also obtained with 3 μM Ifenprodil instead of Ro (data not shown). Taken together, our data suggest that NR2A- and NR2B-type receptors contribute to about 70–80% and 20–30%, respectively, of isolated NMDAR-mediated EPSPs. All blocking effects were statistically significant (p < 0.01) whereas the residual responses after adding both blockers did not differ from zero (p > 0.1).

### Stimulus-induced LTD

The suggested dominant role for NR2A subunits in NMDA-induced LTD seems to contradict previous studies implying a major role for NR2B units in stimulus-induced LTD [18,19]. To resolve this inconsistency we wished to investigate the role of NR2A and NR2B subunits in stimulus-induced LTD in our experimental situation. Since LTD induced by conventional 1–2 Hz stimulation [5,6] was relatively small in our hands we induced LTD by test rate stimulation (0.1 Hz, for a period of 1–2 h) in a low Mg^2+ ^solution. In this situation LTD is facilitated by the easier entry of Ca^2+ ^through NMDARs. Previous work in our lab has shown that this experimental model provides depression down to 40–50% of initial baseline [24]. The depression will be refered to as "slow LTD" in the following.

**Figure 3:**
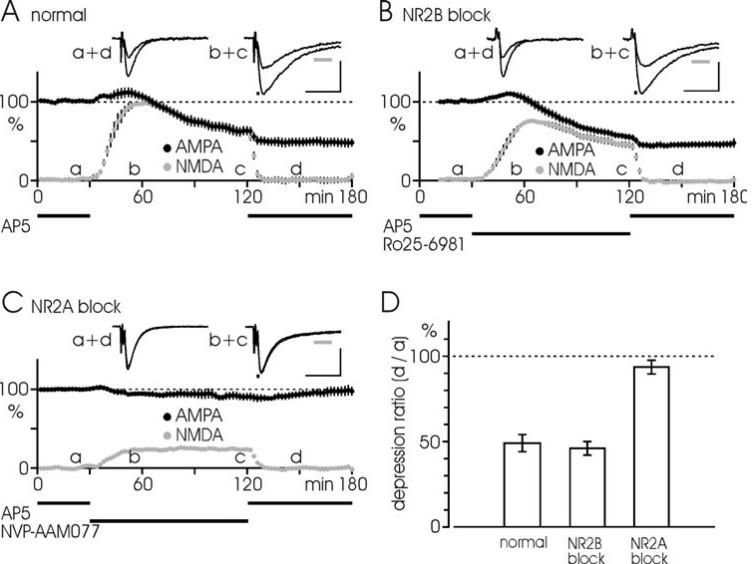
**Roles of NR2B and NR2A subunits in stimulus-induced LTD**. (A) A "slow LTD" is induced by 0.1 Hz test rate stimulation for 90 min in a low Mg^2+ ^solution. The experiment consists of preinduction baseline level in presence of a high concentration of AP5 (50 μM), induction period in AP5-free solution, and established LTD after reintroduction of high AP5. AMPA component (black symbols) and NMDA component (gray symbols) are plotted as functions of time, each point indicating the average in 2 min (n = 8 experiments). (B) A similar result is obtained in the same type of experiment but treated with Ro25-6981 (0.5 μM; n = 8) during the induction period. (C) The "slow LTD" is substantially blocked when NVP-AAM077 (0.4 μM; n = 7) is present under induction in AP5-free solution. (D) Bar diagram shows the "slow LTD" at 30 min after reintroducing AP5, plotted as percentage of the preinduction level under the three different experimental situations in A-C. Data are mean ± S.E.M. Black bars in A-C indicate the duration of drug treatment. Inserts show the EPSP-traces taken at the indicated time points (a-d) and the timing of the measurements for AMPA and NMDA components (0–1.5 ms after fiber volley and at 35–45 ms, respectively; see bars below traces). Calibrations: 0.2 mV, 20 ms.

Fig. 3A illustrates the three stages of a "slow LTD" experiment under normal conditions. Measuring dual EPSPs via an early and a late time window allowed both AMPA and NMDA components to be assessed (see Methods). Initially, a high concentration of AP5 (50 μM) was present to block the NMDA component, the remaining AMPA-EPSP being used to define the preinduction baseline. Next, AP5 was washed out to allow expression of an NMDA response, which caused a gradually developing depression of both AMPA and NMDA components. Finally, AP5 was reintroduced, leading to a depressed level of AMPA-EPSPs by a factor of 49 ± 5% as compared to the initial baseline (n = 8). To test the role of NR2A- and NR2B-type receptors in the induction of this "slow LTD", either NVP or Ro/Ife was introduced in the AP5-free solution (Fig. 3B, C). Our results showed that LTD was uneffected by Ro/Ife, but was largely blocked by NVP, the AMPA-EPSP obtained after the induction period amounting to 45 ± 4% (n = 8, p > 0.1) and 93 ± 4% (n = 7, p < 0.001) of baseline level, respectively. The results are summarized in Fig. 3D.

### LTP, depotentiation and repotentiation

**Figure 4:**
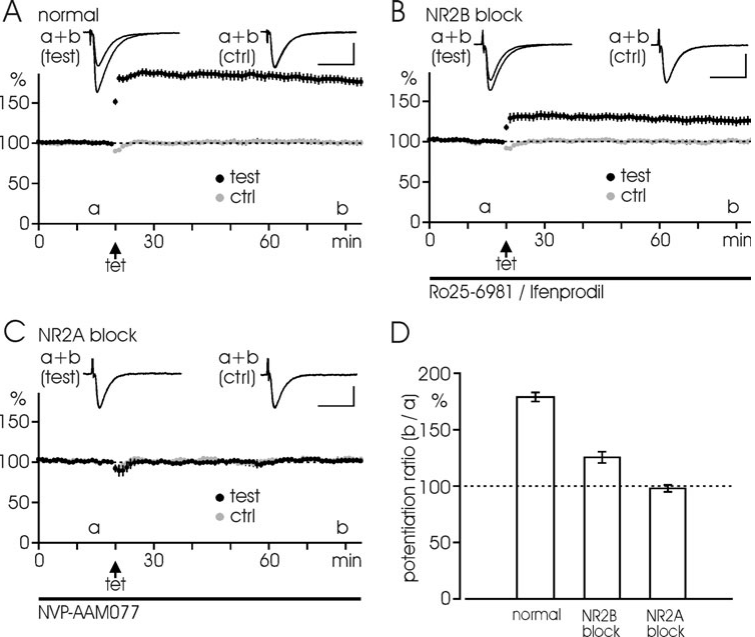
**Roles of NR2B and NR2A subunits in LTP**. (A) LTP induced by three successive tetani (100 Hz, 100 impulses each) in normal solution. The EPSP is potentiated to a near doubling of the initial response. Test pathway (black symbols) and control pathway (gray symbols) are plotted as functions of time, each point indicating the average in 1 min (n = 10 experiments). (B) LTP is partially blocked by Ro25-6981 (0.5 μM; n = 2)/Ifenprodil (3 μM; n = 4) (total n = 6). (C) LTP is fully prevented when NVP-AAM077 (0.4 μM) is present (n = 5). (D) Bar diagram summarizes the data in A-C with mean ± S.E.M. LTP is measured at 60 min after tetanization relative to the initial baseline. Arrows in A-C indicate the tetani. Black bars indicate the duration of drug application. Inserts illustrate EPSP-traces taken at the indicated time points (a, b). Calibrations: 0.5 mV, 20 ms.

Different induction pathways have previously been suggested for LTP versus LTD, so we went on to test this possibility in our experimental situation. LTP experiments were therefore carried out in normal solution in the presence of either NR2A or NR2B inhibitors. Fig. 4A illustrates LTP induced by three successive tetani (100 Hz, 100 impulses each) in the control situation. When measured at 60 min after induction, the EPSP was increased to a level of 179 ± 4% of baseline (n = 10). Consistent with previous findings [18,19], LTP was completely blocked by the prior treatment with NVP (Fig. 4C), the level after tetanization amounting to 98 ± 3% of baseline (n = 5, p < 0.001). Moreover, the degree of LTP was also substantially reduced by Ro/Ife (Fig. 4B), the potentiated level amounting to 126 ± 5% of baseline (n = 6, p < 0.001 for both the blocking effect and the remaining LTP). These results suggest that both NR2A and NR2B subunits contribute to LTP under normal conditions.

**Figure 5:**
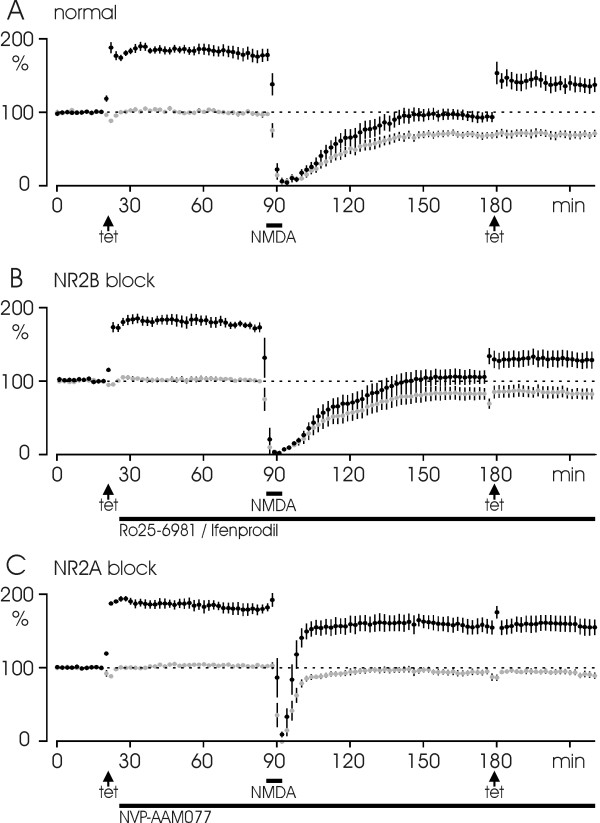
**Roles of NR2B and NR2A subunits in depotentiation and repotentiation after LTP**. (A) LTP is induced by three successive tetani (100 Hz, 100 impulses each) leading to a near doubling of the EPSP. Subsequent application of NMDA (30 μM, 4 min) leads to persistent depression of the control pathway (gray symbols) and depotentiation of the test pathway (black symbols). Secondary tetanization of the test pathway causes a repotentiation, lifting the test responses back to a potentiated level (n = 12). (B) and (C) show similar experiments but treated with Ro25-6981 (0.5 μM; n = 4)/Ifenprodil (3 μM; n = 2) (total n = 6) or NVP-AAM077 (0.4 μM; n = 5) shortly after LTP induction. Both depotentiation and repotentiation are preferentially attenuated by NVP-AAM077 as compared to Ro25-6981/Ifenprodil. Arrows indicate the tetani. Black bars indicate the duration of drug application.

**Figure 6:**
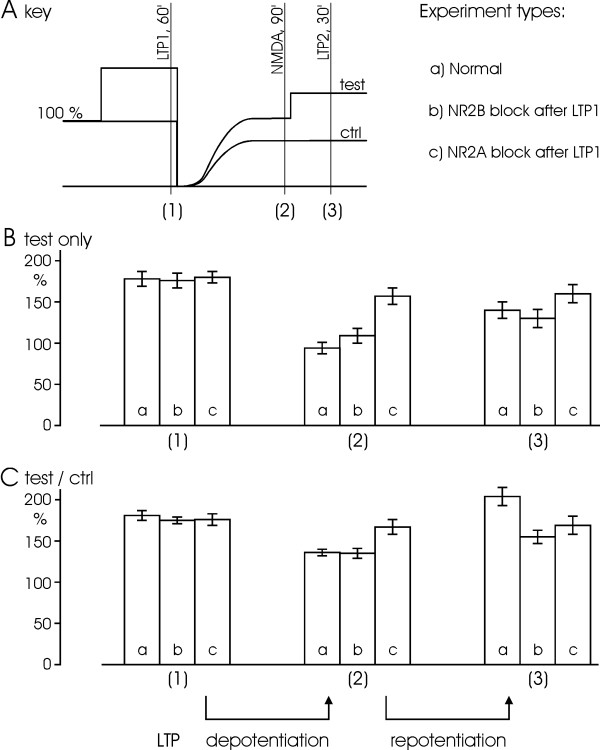
**Summary of depotentiation and repotentiation effects**. (A) Key, defining the measurements used. The experiment model indicates the three types of plasticity, LTP, depotentiation, and repotentiation, subjected to measurements at times (1), (2) and (3), respectively. Additionally, there are three types of experiment with different drug treatment: normal (n = 12), NR2B block (by Ro25-6981/Ifenprodil, n = 6) and NR2A block (by NVP-AAM077, n = 5), denoted a-c and corresponding to panels A-C in Fig. 5 (same primary data used). Since drugs were applied after LTP induction only depotentiation and repotentiation were subjected to the specific subunit blockers. (B) Values of field EPSPs are estimated as test responses in percentage of initial baseline under the three different experimental conditions (a-c) and time points (1–3). (C) Same as B but values are calculated as the ratio between test and control responses for the three types of experiment. Data are expressed as mean ± S.E.M.

We next performed experiments with NMDA application one hour after LTP induction as above, leading to a depression of the naive pathway and a depotentiation of the LTP-treated one (Fig. 5A). A second tetanus was applied to the LTP pathway at 90 min after the NMDA application leading to a repotentiation. Synaptic plasticity was quantified using either the plain test response or test/control ratio (see Materials and Methods), yielding somewhat different values. The properties of these similar but nonequivalent estimates are further considered in Discussion. Under normal conditions, the potentiated level of 178 ± 9% was reduced to 94 ± 7% (n = 12) of the baseline by NMDA application, revealing that depotentiation occurred (Fig. 6B, values marked a). Subsequent tetanization restored the level to 140 ± 10%. When we expressed the values as test/control ratio, the potentiation amounted to 181 ± 6% whereas the depotentiation resulted in 136 ± 4%. Repotentiation finally gave a ratio as high as 204 ± 11% (Fig. 6C, values marked a).

In similar experiments, either NR2A or NR2B inhibitor, i.e. NVP or Ro/Ife, was applied just after the first tetanus (Fig. 5B, C, Fig. 6A values marked b-c), assuring that the drug did not affect induction of the first LTP whereas it had access to all the following plasticity. We found that LTP expression was not affected by either of the blockers, while the subsequent NMDA-induced depotentiation/depression was substantially prevented by NVP (n = 5, p < 0.01) but not Ro/Ife (n = 6, p > 0.1). The second tetanus following NMDA application failed to repotentiate the responses in presence of NVP. Compared to the normal situation, repotentiation only occurred partially under the treatment with Ro/Ife.

### Compensatory role of NR2B subunits in facilitation experiments

**Figure 7:**
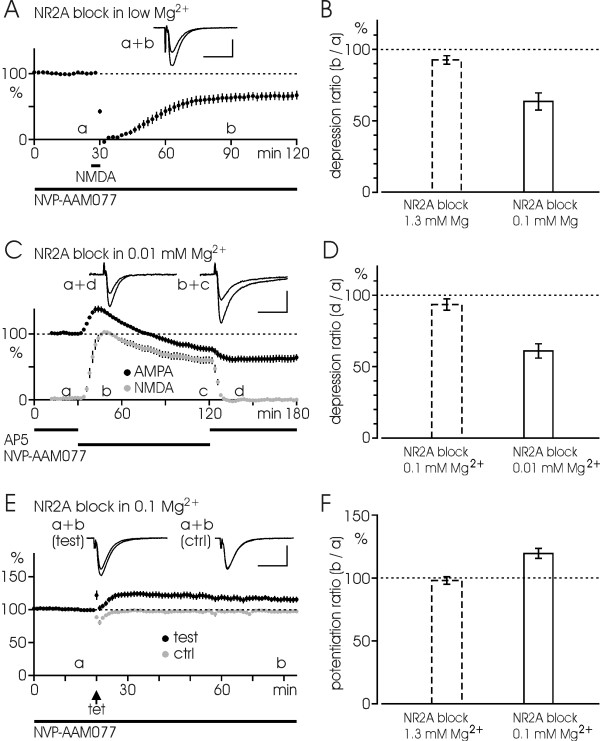
**Facilitated induction of plasticity overrides the blockade by NVP-AAM077**. (A) Blocking effect of NVP-AAM077 on NMDA-induced LTD (see Fig. 1C) is overcome by lowering Mg^2+ ^concentration (0.1 instead of 1.3 mM; n = 6). (B) Bar diagram shows NMDA-induced LTD in different Mg^2+ ^solutions. Dashed bar is control data from Fig. 1D. (C) In a "slow LTD" experiment, similar to that in Fig. 3C, lowering the concentration of Mg^2+ ^in the perfusion solution (0.01 instead of 0.1 mM) helps with the induction of "slow LTD" in presence of NVP-AAM077 (0.4 μM; n = 7). (D) Bar diagram reveals the "slow LTD" induced in different Mg^2+ ^solutions. Dashed bar is control data from Fig. 3D. (E) Similarly, using low Mg^2+ ^solution (0.1 instead of 1.3 mM) in LTP experiments (compare Fig. 4C) enables a small potentiation of EPSPs under inhibition of NR2A subunits by NVP-AAM077 (n = 7). (F) Bar diagram shows LTP induced in different Mg^2+ ^solutions. Dashed bar is control data from Fig. 4D. Data are shown as mean ± SEM. Inserts show the EPSP-traces taken at the indicated time points (a-d). Calibrations: 0.5 mV (A, E), 0.2 mV (C), 20 ms.

It seems from the above that NR2A subunits are essential for the induction of all types of synaptic plasticity we tested here. Alternatively, the blocking effect of NVP could be unspecific and merely due to the fact that the total size of the NMDA response is reduced below a threshold level. The NMDA-EPSP experiment in Fig. 2 illustrates that although NR2A subunits are responsible for a large portion of the EPSP, NMDA responses still have a significant NR2B-mediated part, amounting to 20–30%. Moreover, low concentration of AP5 (5 μM), mimicked the NVP effect on both NMDA responses and "slow LTD", suggesting that there was a threshold effect (n = 3, not illustrated). To test the possibility that the amount of available NMDARs is the crucial factor for inducing synaptic plasticity and so whether NR2B subunits could take over the role of NR2A subunits in presence of NVP, we tried to facilitate induction of some of these NMDA-dependent synaptic plasticities by lowering Mg^2+ ^concentration in the perfusion solution. First we tested the effect of NVP on NMDA-induced LTD in 0.1 instead of 1.3 mM Mg^2+ ^solution. Fig. 7A, B show that NMDA application was able to induce a substantial LTD under this condition, the responses being depressed down to 64 ± 6% of the baseline level (n = 6, p < 0.01), similar to the control experiment (see Fig. 1A). We next induced "slow LTD" in 0.01 mM Mg^2+ ^solution in presence of NVP (reasoning that unblocking might be incomplete with the 0.1 mM Mg^2+ ^otherwise used) and found that activation of NMDARs caused a significant depression in this case as well (Fig. 7C, D), down to 61 ± 5% of baseline (n = 7, p < 0.001). We also carried out additional LTP experiments in low Mg^2+ ^solution (0.1 mM) to boost the induction process. It can be noted that a small LTP was induced under the effect of NVP, 119 ± 4% (n = 7, p < 0.01; see Fig. 7E, F). The results suggest to us that NR2B-type receptors are capable of supporting the induction of these types of synaptic plasticity under the blockade of NR2A-type receptors.

## Discussion

The present study adds information to the contentious issue of NR2A versus NR2B involvement in different forms of NMDA-dependent plasticity. In our case of 12–18 days old rats, there was an overweight for NR2A involvement in all of the plasticities examined: NMDA-induced LTD, "slow LTD", LTP, NMDA-induced depotentiation, and repotentiation. However, under certain conditions we were able to show that NR2B subunits also contribute. The results are consistent with our finding that isolated NMDA-EPSPs contain both NR2A- and NR2B-related portions but with a predominant contribution via NR2A subunits.

### Comparison with previous studies

Though our gathered data on NR2 subunit involvement form a coherent picture, it is at odds with some previous studies demonstrating subunit specificity for certain forms of plasticity. For instance, NR2A inhibitor NVP was found to block hippocampal LTP but not LTD, or even converted LTP into LTD in some situations [18]. Our results agree as far as the blocking effect on LTP is concerned but disagree with respect to the lack of effect on LTD. Some other studies also support the idea of a critical role for NR2A-type receptors in LTP [19,22], especially in adult animals [25]. With respect to NR2B, it can be noted that Ro/Ife was found to selectively block LTD with no effect on LTP [18] whereas in our case the only positive effect of Ro/Ife on synaptic plasticity was a partial blockade of LTP. Another study claiming NR2B involvement in LTD described that Ro/Ife blocked LTD that was obtained in the presence of glutamate uptake inhibitors [19]. However, several other studies concluded that both types of subunits contribute to LTP or LTD [20,21,26,27].

The obligatory role of NR2B in LTD as evidenced by some of the previous work has been questioned by a recent study with contributions from several research groups, which showed that neither Ro nor Ife could prevent LTD [28]. Another recent publication demonstrated that NVP affected both LTP and LTD, but Ro only partially blocked LTP with no effect on LTD [29]. These results are in line with the present findings, although the protocols for inducing LTD are different. Previous work in our lab has demonstrated a comparability of standard low frequency stimulation (LFS)-induced LTD with slow LTD [24], but further investigations will be needed to resolve this issue. The present result of a similar receptor dependence in these two types of LTD seems to support that they may share a common mechanism. The previously reported necessity of a glutamate uptake inhibitor for successful LTD experiments [19], used as an argument for NR2B involvement, has also been questioned since blockade of NR2B-type receptors did not affect the induction of LTD either with or without the drug [29].

While we have no in-depth explanation for the differences among results, it can be noted that experimental parameters often vary between the studies with respect to animal species and age, brain area used, type of preparation and recording type, composition of solutions, temperature and protocols for inducing plasticity. Which of these is most critical is hard to judge but biological factors ought to be basic. For instance, properties of LTP or LTD may differ between various brain regions [30,31]. It is also worth to mention that the experimental animals we used to investigate synaptic plascitity were around two weeks old. At this age, the composition of NMDA receptors on synapses is believed to undergo developmental changes, with the NR2B subunit in the receptor complex being replaced by NR2A subunit. Previous studies have shown that in the neonatal brain, NMDA receptors are composed nearly all by NR2B subunits and that the expression of NR2A starts at 6–10 days postnatal age. The shift from NR2B to NR2A subunit leads to a dominant contribution of NR2A subunits to the NMDA receptor in the adult brain [14,32]. It is a bit unexpected that our 12–18 day rats already have a larger portion of NR2A- than NR2B-type receptors. It seems from our results that the shift process goes rather quick once it starts. Similar tests on one-week postnatal rats should be needed in future studies. In the following we will discuss principles that might govern the triggering of NMDA-dependent synaptic plasticity without delving into details.

### Unified or diversified triggering of plasticity?

Let us consider two extremes, naming them as "unified" and "diversified" triggering. In unified triggering we imagine that sources of Ca^2+ ^act in a cooperative manner, their contributions summing together into a final common pathway. The strength and temporal pattern of the latter signal determine the resulting plasticity. Even so, certain receptor subtypes may be preferentially involved depending on the induction pattern. The summation might be achieved via the total Ca^2+ ^concentration in a common compartment, i.e. the spine. Alternatively, target enzymes near the spine membrane could be sensitive to local entry of Ca^2+ ^but without any systematic relation between the distribution of receptors and enzymes. In diversified triggering, on the other hand, NMDAR subtypes and target enzymes are assumed to have specific distributions in the spine membrane and activation is assumed to occur locally. In the extreme case, each type of NMDAR would be strictly linked, via the proper enzyme, to a certain form of plasticity, e.g. potentiation or depression. The induction pattern would then determine which plasticity is induced by activating a certain subarea of the spine, characterized by a special receptor-enzyme combination.

So where do the present data fit in on the unified-diversified axis? In view of the similar overwight for NR2A in the various forms of plasticity as well as in the composition of isolated NMDA-EPSPs, we conclude that it is near the "unified end". According to this idea, one could imagine that if one receptor is blocked and thus fails to induce a certain form of plasticity, the other type of receptor could take over its role, provided that conditions for inducing plasticity are generally favorable. In fact, this is what was observed in our facilitation experiments where NR2A was blocked by NVP. Thus, in the presence of NVP, both types of LTD as well as LTP could be induced by sufficiently lowering the Mg^2+ ^concentration. Similar, or analogous results have also been reported in other studies. For instance, LTD recorded in cells in ACC, was blocked by either NR2A or NR2B inhibitors suggesting involvement of both receptor types. When cells were depolarized to higher levels, LTD could be induced again despite blockade of one receptor type, demonstrating that both types were actually capable of supporting LTD induction [33].

In another study, cited above, the trigger mode appeared to be at the other extreme, i.e. diversified triggering [18]. In this case, NR2A- and NR2B-type receptors were unable to substitute for each other but functioned as individual, antagonistic controllers of the polarity of the synaptic change. For instance, it was found that blockade of the NR2A-containing population altered the response to LTP-inducing stimuli in such a way that LTD was produced instead of LTP. This was explained by assuming that LTP-inducing stimuli normally activate both NR2A- and NR2B-type receptors, and so might induce both LTP and LTD, but yielding potentiation as net result. According to the proposed scenario, blockade of NR2A-type receptors would then lead to unmasking of the LTD. However, there was no clear explanation of the results in terms of linkage between receptor and enzyme distributions. With respect to receptor distribution, another previous study considered LTD to be induced via NR2B-containing NMDARs located extrasynaptically because of the need for the presence of glutamate uptake inhibitors [19]. Whether, according to this result, the enzymes causing LTD would also have to be located extrasynaptically is not clear but we surmize so. However, it is still questionable how an extrasynaptic LTD-generating mechanism could fit with the well established input specificity of LTD. It seems to us that generation of LTP or LTD in local membrane regions would have to occur within the spine membrane for the simple reason of input specificity. Notably, it has been demonstrated that both NR2A-type and NR2B-type receptors exist both intra- and extrasynaptically [16].

As mentioned, our data support the idea of unified triggering as several other studies do, even though details in the blocking profiles of NVP and Ro/Ife differ [20,21,27,33]. One of our results that might be hard to explain within this framework is the substantial blockade of LTP by Ro/Ife, despite the fact that NR2B-containing receptors contributed to a relatively small fraction of the NMDA response. In fact this blockade could be on account of diversified triggering to some extent. However, a specific coupling between NR2B receptors and LTP-generating enzymes seems unlikely in view of the fact that NVP was an even more effective blocker of LTP. It is also notable that both blockers had larger effects on LTP than on any of the two kinds of depression. Hence, it could be that different forms of plasticity are differentially sensitive to general reduction of the NMDA response, depending on the functional connection between different induction patterns and specific enzymes.

Even LTP by itself appears to have different sensitivity to NMDAR blockade depending on different induction protocols. For instance, it was shown that tetanus-induced LTP in the ACC was partially blocked, whereas theta-burst-induced LTP was fully blocked by either NVP or Ro [21]. Another recent study demonstrated that tetanus-induced LTP was reduced by either NVP or Ro whereas pairing-induced LTP was unaffected [27]. Moreover, LTP has been reported to be more sensitive than LTD to partial block by AP5 [[18], see also discussion in [20]].

### Specificity of NR2 blocking drugs

This study is based on using NMDAR blockers with specificity for either NR2A- or NR2B-containing heteromers. With respect to possible cross-effects, the NR2B inhibitors Ro and Ife are trouble-free since they are more than hundredfold (Ife) or thousandfold (Ro) more selective for NR2B as compared to NR2A [34,35]. The published data suggest that the concentrations of 3 μM (Ife) or 0.5 μM (Ro) that we used are adequate. Regarding NVP, experiments on recombinant human NMDARs expressed in oocytes indicated a reasonably high selectivity of the drug, about 130-fold for NR2A versus NR2B [36]. In contrast, similar experiments expressing rodent NMDA receptors in either oocytes or HEK-293 cells revealed a smaller ratio, around 13–14-fold [29,37]. NVP at 0.4 uM concentration, i.e. the same as used in the present study, was shown to block most of the response of NR2A-expressing cells, as measured by Ca^2+ ^imaging, down to a few percent of control [29]. However, the response of NR2B-expressing cells to the same NVP concentration was also affected, being decreased by about 35–40% [29]. Assuming that similar cross-effects applied to our case, the obtained value of 19% remaining NMDA response after NVP treatment actually implies around 30% in a theoretical case without cross-effect. This value fits well with our finding that 68% of the NMDA response remained after Ro/Ife treatment, i.e. a reduction by 32%. This nice agreement may be partly a chance fit since there are complicating factors, such as the unknown blockade of mixed NR1/NR2A/NR2B heteromers, and a higher cross-reaction reported in other work on HEK293 cells [20]. Additionally, a study of NVP effects in mutant NR2A-deficient mice also reported that the drug was effective in blocking NR2B-receptors, and argued for careful interpretation of data when using NVP [26]. To some extent, the limited selectivity of NVP may weaken our conclusion that NR2B receptors can support LTP/LTD under NVP blockade in facilitation experiments, considering that residual NR2A current would not be negligible compared to the remaining NR2B response. However, the general idea of NR2A dominanting over NR2B remains valid. Moreover, our comparison between blockade of responses and blockade of synaptic plasticity ought to be unaffected by cross-effects, since both cases would be influenced in a similar manner.

### Properties of depotentiation

Depotentiation refers to the erasure of LTP by stimulation applied after the induction. Previous studies have shown that depotentiation can be induced by stimulation similar to that used for inducing LTD [38]. Induction of depotentiation is generally believed to be NMDA-dependent although there are also reports on NMDA-independent depotentiation, e.g. selectively involving AMPA receptors [39-41]. In the present case we studied depotentiation induced by application of NMDA and examined its sensitivity to NR2A and NR2B inhibitors, since not much attention has been paid to this issue previously. It can also be noted that depotentiation has mostly been described in terms of changes of the tetanized pathway, whereas in the present study, we also considered the relation between two pathways as a suitable estimate for studying depotentiation and repotentiation. Although the used test/control ratio is "contaminated" by the LTD of the control pathway, it has the advantage that any unspecific plasticity unrelated to LTP or LTD is eliminated. As described below, the results can be more easily understood by hypothesizing the existence of a superimposed, unspecific depression.

Let us consider some essential features of depotentiation that emerged from our results in normal solution. It can be noted that NMDA application caused depotentiation independent of the estimate used, i.e. test vs. test/control, even though the values differed. The estimates were taken under stable conditions after full recovery of responses after NMDA application. However, in view of the similar time courses of the recovery of test and control pathways (see Fig. 5A) we hypothesize that both NMDA-induced LTD and depotentiation were established early after NMDA application but were confounded by the recovery from extinction, constituting an early transient depression that attenuated test and control responses in a parallel manner. Interestingly, assuming that this hypothesized depression also has a stable part may help us to understand the "annoying fact" that repotentiation after NMDA-induced depotentiation is only partial [9], as also confirmed by the present work. A simple explanation would be that LTP was not fully depotentiated and that the apparent full depotentiation was due to a mixture of true depotentiation and the hypothesized depression. In accord with this view, when using relative estimates in terms of test/control, the original LTP was fully reinstated or even overexpressed by the second tetanus. The latter reflects the fact that comparison was made with a depressed pathway. Further work is needed to interrogate the idea of multiple depression mechanisms.

When it comes to the role of NR2A and NR2B subunits, we found that the NMDA-induced depotentiation was largely blocked by NVP but was little influenced by Ro/Ife, suggesting predominant involvement of NR2A-containing NMDARs. This agrees with a previous study on depotentiation induced by "conventional" LFS [19]. The major contribution of NR2A subunits is also in accord with with our primary finding that NVP but not Ro/Ife blocked NMDA-induced LTD, confirmed by the control pathway data in depotentiation experiments. It so appears that the induction properties of depotentiation and LTD are similar, at least with respect to NMDAR subtype involvement. As might be expected, repotentiation after NMDA-induced depotentiation was fully blocked by NVP whereas Ro/Ife had only a partial effect. The reasoning is that repotentiation would be equivalent to de novo potentiation, except for a possible difference in baseline level. However, in the case of NVP, the fact that this baseline (before the second tetanus) was largely devoid of depotentiation provides a complementary explanation for the lack of repotentiation. In future studies, a set of similar repotentiation experiments, but with NVP applied after the NMDA-induced depotentiation, should give us a better view of how NVP affects repotentiation. Futhermore, it is not known whether larger repotentiation would be possible in NVP solution under conditions of facilitated induction, in analogy with our results on LTP and LTD.

It can also be observed that the level of LTP after repotentiation in Ro/Ife solution was substantially less than in the normal case. This was especially noticeable when using relative estimates (test/control). As discussed above, relative estimates suggest that full recovery of LTP may be possible after repotentiation. Hence, it remains to find out whether repeated tetanization in the presence of Ro/Ife, after NMDA application, would be able to fully restore the initial level of LTP.

## Conclusion

By studying the involvement of NMDAR-subunits in several forms of NMDA-dependent synaptic plasticity we arrive at some general ideas about the integration of postsynaptic signals in the Schaffer-collateral pathway of 2-week-old rats. We conclude that NR2A- and NR2B-containing NMDARs mediate most of the NMDA response, with NR2A dominating over NR2B. Similarly, both NR2A- and NR2B-type receptors can participate in triggering synaptic plasticity, but NR2A showing a foremost contribution to all the forms of synaptic plasticity tested here. Our results support a scenario in which Ca^2+ ^influx, entering the dendritic spine via different NMDAR heteromers, sums into a "final common pathway" that controls synaptic plasticity by its magnitude and temporal pattern regardless of the source.

## Methods

### Slice preparation and maintenance

Hippocampal slices were prepared from Sprague-Dawley rats aged 12–18 postnatal days, including both males and females. Animal handling complied with the guidelines of the Swedish Central Council for Laboratory Animals and was approved by the local Ethical Committee for Animal Research. After initial isoflurane (Forene) anesthesia and decapitation, the brain was removed and placed in an ice-cold artificial cerebrospinal fluid (ACSF) solution composed of (in mM): 119 NaCl, 2.5 KCl, 2 CaCl_2_, 2 MgCl_2_, 1 NaH_2_PO_4_, 26 NaHCO_3 _and 10 glucose. Transverse hippocampal slices (400 μm) were cut using a vibrating tissue slicer (Campden Instsruments) and transferred to the holding chamber, containing the same ACSF as above and gassed with 95% O_2_, 5% CO_2_. Slices were stored in the holding chamber at room temperature for at least 1 h before the experiments. For field potential recording, slices were individually transferred to one or several "submerged type" recording chambers, perfused at 30–32°C by the same solution and gas components as above, except that Ca^2+ ^was 2 or 2.5 mM, and Mg^2+ ^was 1.3, 0.1 or 0.01 mM depending on needs. Perfusion speed was 1.5 ml per min. AMPA EPSPs were normally recorded in ACSF with 2.5 mM Ca^2+ ^and 1.3 mM Mg^2+^. Composite AMPA-NMDA EPSP recording used 2.0 mM Ca^2+^, 0.1 mM (or 0.01 mM) Mg^2+ ^to facilitate the NMDA response. In this case, a low concentration of CNQX (0.5–1 μM) was also present to partially block the AMPA response, leading to a balanced mixture of AMPA and NMDA components. Isolated NMDA-EPSPs were obtained with 2.0 mM Ca^2+^, 0.1 mM Mg^2+^, and 10 μM CNQX to fully block the AMPA component.

### Stimulation and recording

Field excitatory postsynaptic potentials (EPSPs) were recorded from the CA1 apical dendritic layer using a glass micropipette filled with perfusion solution (ACSF) or 1–3 M NaCl, 2–5 Mohm resistance. Stimulation, usually repeated once per 10, 30 or 60 s, was delivered as 0.1 ms negative constant current pulses (10–50 μA), via one or two monopolar tungsten electrodes to Schaffer-collateral axons. In two-pathway experiments, the stimulating electrodes were positioned symmetrically on each side of the recording elcctrode, allowing alternating activation of two nonoverlapping sets of synapses. The rate of stimulation was maintained throughout the experiment except for periods of LTP induction. LTP was induced by three successive tetani (100 Hz, 100 impulses each) repeated with an interval of 5 s in between the trains. NMDA-induced LTD was acheived by a brief (4 min) application of NMDA (30–40 μM). In "slow LTD" experiments, the stimulus frequency was kept constant at 0.1 Hz and NMDA-dependent induction was brought about by pharmacological unblocking of NMDARs for 1–2 h by help of a low Mg^2+ ^solution (usually 0.1 mM). Stability of recordings was verified for at least 1 h before beginning the actual experiment.

### Data Analysis

Signals were amplified, filtered and transferred to a PC clone computer for on-line and off-line analysis by specially designed electronic equipment (based on an Eagle Instruments multifunction board) and own developed computer software. AMPA EPSPs, as well as the AMPA component of composite EPSPs, were measure using a time window of 0–1.5 ms after the fiber volley. The NMDA component of composite EPSPs was measured with a 35–45 ms window whereas isolated NMDA-EPSPs were measured using a window of 0–5 ms after the fibre volley. Measurements were calculated by integrating the EPSP curve along the specified time window after subtraction of the prestimulus baseline, yielding the area under the curve. This measure can also be viewed as the "initial amplitude" and has similar properties as "initial slope". Values were corrected by subtracting the corresponding measurements of the nonsynaptic potential, obtained after total blockage of the EPSP by 10 μM CNQX and 50 μM AP5. In most cases, the traces used for illustration were also corrected by the nonsynaptic potential. Synaptic plasticity was generally estimated as ratio between post- and pre-induction values after averaging responses for 5–10 min periods. In two-pathway experiments, the ratio between pathways was sometimes used. In some experiments with NMDA component recording, there was no preinduction baseline available to be used as 100%. In this case the maximum level was generally used as 100%. Additional scaling of this level was performed in experiments with NMDA subunit blockers to include the effect of the blocker. Data are presented as mean ± S.E.M. Statistical comparisons were made using Student's *t*-test. Statistical significance was set at p < 0.05.

### Drugs

CNQX and AP5 were obtained from Tocris Cookson, UK; Ro25-6981 and Ifenprodil were from Sigma Chemicals Co., MO, USA. NVP-AAM077 was generously supplied by Novartis Pharmaceuticals. Prefabricated stimulating electrodes were obtained from World Precision Instruments, FL, USA, type TM33B.

## Abbreviations

AMPA: α-amino-3-hydroxy-5-methyl-4-isoxazolepropionic acid

AP5: D(-)-2-amino-5-phosphonopentanoic acid

CNQX: 6-cyano-7-nitroquinoxaline-2,3-dione

EPSP: excitatory postsynaptic potential

Ife: Ifenprodil

LTD: long-term depression

LTP: long-term potentiation

NMDA: N-methyl-D-aspartate

NMDAR: NMDA receptor

NVP: NVP-AAM077

Ro: Ro25-6981

## Authors' contributions

RL conceived the study, carried out most of the experiments and participated in writing the manuscript. FSH performed some of the experiments and provided useful discussion. AKA helped with some experiments. HW designed the experiments, supervised the study, helped with analysis of data, prepared the figures and involved in editing the manuscript. All authors read and approved the final manuscript.
